# Attitude and perception of medical interns about antimicrobial resistance: a multi center cross-sectional study in Ethiopia

**DOI:** 10.1186/s13756-018-0443-9

**Published:** 2018-12-04

**Authors:** Amanual Getnet Mersha

**Affiliations:** School of Medicine, College of Medicine and Health Sciences, University of Gondar, P.O. Box: 196, Gondar, Ethiopia

**Keywords:** Antimicrobial, Antimicrobial resistance, Medical intern, Education, Ethiopia

## Abstract

**Background:**

Antibiotic resistance is a global burden and reduction of antimicrobial resistance requires change in antimicrobial prescribing behavior of health workers. The current study aimed to evaluate the attitude and perception of Ethiopian medical interns towards antimicrobials resistance.

**Methods:**

A multi center cross-sectional institutional based survey was conducted from August 2016 to October 2016 among medical interns in Ethiopia. Data entered and analyzed using Statistical Package for Social Sciences program (SPSS) software version 21.0 for Windows. Descriptive statistics, chi-square test and binary logistic regression analysis were used and statistical significance was set at *P-value* < 0.05 as a cut of point.

**Results:**

Out of the 278 questionnaires distributed, 270 were returned completed giving a response rate of 97.1%. Most of the participants 256 (94.8%) wants to receive further education about antimicrobial stewardship. Chi-square test showed a significant difference between institutions about interns’ attitude and perception concerning over usage of antimicrobials in their institutions; description of correct antimicrobial coverage; handling patients who demands antimicrobial therapy that is not indicated and finding reliable sources of information to treat infections (*P-value* < 0.05). Attaching at internal medicine wards during the survey and receiving antimicrobial stewardship training during the past 6 months were found to improve their attitude significantly by 2.68 and 3.48 times respectively.

**Conclusions:**

The current study demonstrates an enormous desire of medical interns for further education about antimicrobial stewardship. Hence, it is strongly recommended to provide a comprehensive, regular, standard and up to date educational training in all medical institutions for our future prescribers. Medical institutions and stakeholders are recommended to advocate curriculums and policies that build up antimicrobial stewardship programs.

## Background

Antimicrobials are the most commonly prescribed drugs in developing as well as many developed countries to treat and control infectious disease [1, 2]. Inappropriate use of antibiotics results in bacteria resistant to antibiotics in the community and hospital [3]. Antibiotic resistance is a worldwide problem, with a negative consequence on patient treatment outcomes [4]. Furthermore, patients infected with drug resistant bacterial infection need more expensive intervention, so antimicrobial resistance results in increased health care costs to families and societies [5]. Reducing the burden of antimicrobial resistance is highly challenging in low-income countries than developed countries due to high prevalence of infection, irrational uses of antimicrobials, over-the-counter availability of antibiotics and lack of clinical microbiology laboratories for antimicrobial susceptibility testing in the developing countries [6].

In most countries health care system physicians are responsible for prescribing antibiotics. However, junior medical doctors feel unprepared for the complexity of antibiotic prescribing in their daily practice [7]. Several international studies have identified areas of lack of confidence and gaps in antibiotic prescribing knowledge, and have shown that medical students perceive a need for further education and training on antibiotic prescribing and antimicrobial stewardship program [8]. Education can influence prescribing behavior and can provide knowledge that will enhance the acceptance of stewardship strategies among health care practitioners [9]. In most countries education system, there are no enough antimicrobial resistance education programs at undergraduate level and are still being conducted at the postgraduate level in healthcare institutions [10]. Evidence suggests that a multifaceted approach is favored at improving the organization of the healthcare system and changing physicians’ prescribing behavior and practice [11].

Reduction of antimicrobial resistance requires change in antimicrobial prescribing behavior of health workers, antimicrobial prescribing patterns and physicians’ behavior towards the magnitude of antimicrobial resistance problem [12]. The World Health Organization has recommended training for medical undergraduates students regarding appropriate prescription of antimicrobials [13]. It is necessary that our future doctors are better equipped with better knowledge, attitude towards antimicrobial use and resistance [14]. Unlike the senior physicians or infectious specialists who have a large amount of experience and knowledge in infection treatment, the junior doctors usually have limited knowledge and skills to reduce the potential risk of antimicrobial resistance [15]. Therefore, antimicrobial stewardship efforts should be made to standardize the prescribing behaviors of medical inters [16].

In Ethiopia, few studies were conducted concerning the prevalence and associated factors of inappropriate use of antibiotic [17, 18]. However, there is no study conducted in Ethiopia to assess the attitude and perception of medical inters towards antimicrobial resistance. Hence, the purpose of this study is to assess the attitude and perception of medical inters on antimicrobial resistance at teaching hospitals in Ethiopia. The information generated in this study would be instrumental in planning and implementing preventive and control interventions on antimicrobial resistance and applying antimicrobial stewardship program at regional and national levels.

## Methods

### Study design and setting

A multi center cross-sectional institutional based survey was conducted from August 2016 to October 2016 among interns working in three teaching hospitals (hospitals that provide medical education to future health professionals) in Ethiopia. All of the three medical institutes included in the study are tertiary level teaching hospitals with a graduate and post graduate residency programs.

### Sample size determination and procedure

A two-stage sampling procedure was employed to select participants of the study. In the first stage, all 11 institutions with practicing interns in Ethiopia were listed and three institutions were randomly selected. In the second stage, the list of practicing interns were taken and participants selected by using a random selection method. To determine the sample size, a single population proportion formula was used with the assumption of 95% confidence interval, 5% margin of error, 50% estimated perceived need of further training about antimicrobial stewardship, and 10% for possible non-response was taken to determine a final sample size of 278. The total sample was proportionally allocated based on the number of interns in each institution (Institution A - 148, Institution B - 151, and Institution C - 145).

### Data collection and management

After a thorough review of relevant literatures a structured questionnaire validated to our setting was prepared and reviewed by subject experts [19–22]. The questionnaire is divided into three parts. The first part assesses participants’ characteristics including gender, age, AMS training in the last 6 months (trainings that focus on antimicrobial prescribing, appropriate use of antimicrobials, and antimicrobial resistance), and ward distribution during filling the questionnaire. The second part assesses interns’ attitude and perception about antimicrobial prescribing and antimicrobial resistance. A 5-point Likert scale, whose responses range from ‘strongly agree’ to ‘strongly disagree’ was used to assess their attitude and perception. The third section assessed interns’ attitudes and perceptions about their education level about appropriate antimicrobial use and antimicrobial stewardship. In this section also a 5-point Likert scale, whose responses ranged from ‘very good’ to ‘very poor’ was used. The questionnaire was pretested on 5% of the sample size prior to the real data collection, which was excluded from the final study, and appropriate modifications were also done.

### Statistical analysis

After checking the completeness, the three institutions were de-identified and recoded as “A”, “B”, and “C”. Answer to questions that used a five point Likert scale was condensed in to two classes (agree/strongly agree and neutral/disagree/strongly disagree) and (good/very good and neutral/poor/very poor). Data entered and analyzed using Statistical Package for Social Sciences program (SPSS) software version 21.0 for Windows. The analysis of answers for other questions involved descriptive quantitative statistics described using frequency and percentage. Chi-square tests were applied to compare between sites and statistical significance was set at *P* < 0.05 as a cut of point. Binary logistic regression analysis was employed to assess possible associations between different variables and intern’s attitude and perception. To investigate possible associated factors with attitude towards antimicrobial stewardship, agree or strongly agree to four or more of the following six attitude questions (inappropriate use of antimicrobials causes antimicrobial resistance; strong knowledge of antimicrobials is important in my medical career; appropriate use of antimicrobials will reduce problems with antimicrobial-resistant organisms; inappropriate use of antimicrobials can harm patients; poor infection-control practices cause spread of antimicrobial resistance; prescribing broad-spectrum antimicrobials when equally effective narrower-spectrum antimicrobials are available increases antimicrobial resistance) were used as a cut-off point to say “good attitude”. Analysis was done by a person who was not involved in the coding process of institutes.

### Ethical consideration

This study was approved by the ethical review board of University of Gondar. Written informed consent from the participants was also received before conducting this study. The obtained information from the participants was kept confidential.

## Results

### Characteristics of the participants

Out of the 278 questionnaires distributed to three centers (Institution A - 93, Institution B – 94, and Institution C - 91), 270 were returned completed giving a response rate of 97.1%. The mean age of participants was 25.21 ± 3.74 years old. Almost one-third of participants 89(32.9%) were female and the remaining 181(67.1%) of respondents were male. Among the participants 31(11.5%) had received antimicrobial stewardship training in the past 6 months before the study. Among the participants 71(26.3%) are working at internal medicine wards; 66(24.4%) are working at pediatrics wards; 64(23.7%) are working at gynecology and obstetrics wards and 69(25.6%) are working at surgical wards.

### Attitudes and perceptions about antimicrobial prescribing and resistance

As illustrated in Table 1, 266 (98.5%) of participants perceived that acquiring strong knowledge of antimicrobials is important in their future medical career. The majority of medical interns included in the current study 256 (94.8%) wants to obtain further education regarding antimicrobials resistance. Majority of medical interns believed there are over usage of antimicrobials nationally 245 (90.7%) and 252 (93.3%) of study participants perceive antimicrobials resistance as a national concern. Three-fourth of participants 197 (72.9%) perceived that antimicrobials are being overused in their hospital with differences between educational institutes range from 53 to 89%. Chi-square test was performed to evaluate whether these proportion differences among centers are significance and it was found to be significant (*P - value* of 0.032). About one-third of the interns 82 (30.4%) have a perception that new antimicrobials will be developed that will alleviate the problem of antibiotics resistance with a significant group differences among educational institutions that ranges between 19.3 and 39.8% (*P - value* of 0.021) (Table 1).

**Table 1:** Medical Interns’ attitudes and perceptions about antimicrobial prescribing and resistance

Variables	Agree/strongly Agree N (%)	*P - value*
Inappropriate use of antimicrobials causes antimicrobial resistance	261 (96.6%)	0.071
Strong knowledge of antimicrobials is important in my medical career	266 (98.5%)	0.321
I would like more education on antimicrobials resistance	256 (94.8%)	0.092
Appropriate use of antimicrobials will reduce problems with antimicrobial-resistant organisms	258 (95.5%)	0.135
I would like more education on the appropriate use of antimicrobials	254 (94.0%)	0.219
Antimicrobials are overused nationally	245 (90.7%)	0.319
Inappropriate use of antimicrobials can harm patients	261 (96.6%)	0.424
New antimicrobials will be developed in the future that will keep up with the problem of antibiotics resistance	82 (30.4%)	0.021^*^
Poor infection-control practices cause spread of antimicrobial resistance	244 (90.4%)	0.950
Antimicrobials are overused in our hospitals	197 (72.9%)	0.032^*^
Prescribing broad-spectrum antimicrobials when equally effective, narrower-spectrum antimicrobials are available increases antimicrobial resistance	258 (95.5%)	0.670
Antimicrobial resistance is a significant problem in our hospitals	258 (95.5%)	0.561
Antimicrobial resistance is a significant problem nationally	252 (93.3%)	0.862

^*^Refers to statistically significant differences (*p-value* < 0.05) in the percentages of different teaching hospitals using χ2 test

### Perceptions of medical interns about their educational level towards antimicrobial stewardship

As demonstrates in Table 2, 71 (26.3%) of medical interns believe they can describe the correct range of coverage for antimicrobials. Chi-square test performed among the three institutional groups included in this multicenter study to evaluate whether these proportion differences among centers are significance and was found to be significant (*P - value* of 0.014, range 18.2–31.1%). Among all participants 190(70.4%) interns feel incompetent to handle a patient who demands antimicrobial therapy while it is not indicated with a significant group differences (*P - value* of 0.033, range 56.9–81.2%). Among the participants 119 (44.1%) of interns believe they can found reliable sources of information to treat infections with significant differences between institutes (*P - value* of 0.039, range 31.9–50.9%) (Table 2).

**Table 2:** Medical Interns’ perceptions of their education regarding antimicrobial stewardship

Variables	Good/Very Good (%)	*P- value*
Describe the correct range of coverage for antimicrobials	71 (26.3%)	0.014^*^
Change from intravenous to oral antimicrobials and vice verse	62 (22.9%)	0.821
Handle a patient who demands antimicrobial therapy that is not indicated	80 (29.6%)	0.033^*^
Escalate antimicrobial therapy	98 (36.3%)	0.662
Select the best antimicrobial for a specific infection	95 (35.2%)	0.431
Find reliable sources of information to treat infections	119 (44.1%)	0.039^*^
When to start antimicrobial therapy	133 (49.3%)	0.652
Basic mechanisms of antimicrobial resistance	195 (72.2%)	0.238

^*^Refers to statistically significant differences (*p-value* < 0.05) in the percentages of different teaching hospitals using χ2 test

### Factors associated with better attitudes and perceptions towards antimicrobial stewardship

Logistic regression analysis was employed to assess possible associations between different variables and intern’s attitude and perception. According to the results from bivariate logistic regression, there were statistically significant differences in age, sex, ward attachment during the study period and receiving antimicrobial stewardship training. Variables that were significantly associated with the intern’s attitude and perception in the bivariate analysis (*p*-value < 0.2) were further examined in multivariate logistic regression. Accordingly, ward attachment during the study period and receiving antimicrobial stewardship training in the past 6 months remained to be significant in the multivariate logistic model. The odds of having good attitude and perception about antimicrobial stewardship was found to have 2.68 times higher among interns attached at internal medicine wards during the data collection than interns attached at surgical wards (AOR = 2.68, 95% CI = 1.73–7.19, *p*-value - 0.032). Receiving antimicrobial stewardship training during the past 6 months was found to be 3.48 folds associated with good attitude towards antimicrobial stewardship(AOR = 3.48, 95% CI = 2.97–5.31, *p*-value - 0.006) (Table 3).

**Table 3:** Factors Associated with better attitudes and perceptions about Antimicrobial stewardship

Variable	N (%)	AOR(95% CI)	*P-value*
Age (25.21 ± 3.74 years old)			0.091
Sex			0.280
Male	181(67.1%)		
Female	89 (32.9%)		
Ward attachments during the study		2.68 (1.73–7.19)	0.032^*^
Internal Medicine	71 (26.3%)		
Pediatrics	66 (24.4%)		
Gynecology and Obstetrics	64 (23.7%)		
Surgery	69 (25.6%)		
Interns who received AMS training during the past 6 months		3.48 (2.97–5.31)	0.006^*^
Yes	31 (11.5%)		
No	239 (88.5%)		

^*^ Refers to statistically significant association

## Discussion

Antimicrobial resistance imposes a substantial public health burden all around the globe [1]. In the current study, the vast majority of medical interns believed that antimicrobial resistance is a main concern in their hospitals as well as in the nation at large. A considerable number of participants reported over usage of antimicrobials nationally. Interestingly, a quarter of participants reported absence of antimicrobial over usage in their hospitals with a statistically significant difference between the participating institutions (*p-value*, 0.032). This result is in line with a study conducted in seven European medical schools which reported that majority of the participants (92%) believe antimicrobial resistance as a national concern [23]. Similarly, a study done at three US medical schools also reported majority (94%) of participants believe presence of antimicrobial usage nationally and 65% believe over usage in their hospital with substantial difference among medical schools [19].

Substantial number of participants perceived that strong knowledge of antimicrobials is important in their medical career and they would like to gain further education on antimicrobials use and resistance. Other studies conducted among medical students and junior physicians in china, USA, and Europe also come up with similar findings [14, 19, 23]. Less than one third of interns believed new antimicrobials will be developed that will alleviate the problem of antibiotics resistance with significant differences among educational institutes (*p-value*, 0.021). A multi-center study done in central China also reported a significant difference among participating medical schools regarding development of new antimicrobials [14]. .This could be due to lack of a standardized, comprehensive, and up to date training concerning antimicrobial stewardship.

One of the main components of reducing antibiotic resistance is administering a specific antimicrobial to cover a specific organism. This study demonstrated that only a quarter of interns believe they can describe the correct range of coverage for antimicrobials with a significant difference between institutions (*p-value*, 0.014). This finding is comparable with study done in other countries [14, 19]. Furthermore, less than a quarter believes they know when to switch from intravenous to oral antimicrobials and vice verse. Proportionally higher number of participants lacks confidence selecting the best antimicrobial for a specific infection. Medical schools are recommended to develop a locally validated protocols concerning switching from intravenous to oral antimicrobials and vice verse [24–26].

.Having a basic understanding of the mechanisms for the development of antimicrobial resistance and finding a reliable source of information to treat infections is vital to challenge the evolving issue of antimicrobial resistance. In the current study, almost three fourth of participants believe they have basic knowledge of mechanisms of antimicrobial resistance. Less than half of the interns believe they can find reliable sources of information to treat infections with statistically significant variation among the different sites (*p-value*, 0.039). This difference may be partially explained by variation in internet access and difference in curriculum interpretation between institutions.

Handling patients who demands antimicrobial therapy without any indication is also vital to reduce the inappropriate use of antimicrobials. Substantial number of participants lacks confidence on how to handle a patient who demands antimicrobial treatment while it is not indicated with a statistically significant difference among study institutions (*p-value,* 0.033). The significant difference between institutions may be due to differences in type of clients visiting the institutions [9, 27]. In Ethiopia medical students receive a detailed lectures and seminars concerning antibiotics, mechanisms of antibiotic resistance and preventing strategies of resistance as part of Pharmacology course in their pre-clinical year. A multi center survey conducted in South Africa illustrated the existence of a huge training gap among final year medical students [28]. The odds of having good attitudes and perceptions about antimicrobial resistance among interns attached at internal medicine wards were found to have 2.68 times better attitudes and perceptions about antimicrobial resistance than interns attached at surgical wards. It could be due to differences in the types of patients admitted at medical and surgical wards. Most of the patients admitted at medical wards in developed countries have infectious disease. Hence, interns at medical wards tend to know more about antimicrobial resistance. Interns who received antimicrobial stewardship training during the past 6 months tends to have better attitudes and perceptions about antimicrobial resistance then those interns who didn’t receive training in the 6 month period before the study. This result is in line with other studies conducted among a Varity of communities. In one randomized controlled trial conducted among general practitioners of France, standardized educational seminar on antibiotic improve the irrational prescription of antimicrobials [29, 30].

### Strength and limitation of the study

This study highlights an area of antimicrobial stewardship where there is lack of literature in Ethiopia. Yet, the survey has some limitations that should be taken into account while interpreting the results. As the study was a cross-sectional survey conducted in only three institutions, caution should be exercised when generalizing the findings. Even with the above limitations, this survey has significant implications for combating antimicrobial resistance.

## Conclusion and Recommendation

The current study demonstrates an enormous desire of medical interns for further education about antimicrobials stewardship. Medical interns believe the gained education concerning appropriate antimicrobial use and antimicrobial stewardship is not enough. Based on our finding we recommend that medical interns should acquire a comprehensive, regular, scheduled and up to date educational training on antimicrobial stewardship so as to answer their educational demand and to combat the issue of antimicrobial resistance by endowing the young physicians with sufficient knowledge. It is recommended to medical institutions and other stakeholders to advocate curriculums and policies that build up antimicrobial stewardship programs in the country. An arrangement should be made that can prepare and disseminate locally validated antimicrobial use guidelines with commonly available antimicrobials in the institution.

## Abbreviations

AM: Antimicrobial
AMR: Antimicrobial Resistance
